# Separate and combined effects of boat noise and a live crab predator on mussel valve gape behavior

**DOI:** 10.1093/beheco/arad012

**Published:** 2023-04-08

**Authors:** Jeroen Hubert, A Daniëlle van der Burg, Rob Witbaard, Hans Slabbekoorn

**Affiliations:** Institute of Biology Leiden, Leiden University, The Netherlands; Institute of Biology Leiden, Leiden University, The Netherlands; NIOZ Royal Netherlands Institute for Sea Research, Dept. Estuarine and Delta Systems, Yerseke, The Netherlands; Institute of Biology Leiden, Leiden University, The Netherlands

**Keywords:** anthropogenic noise, bivalve, *Carcinus maenas*, crab, mussel, *Mytilus*, predator–prey interaction sound

## Abstract

Noisy human activities at sea are changing the acoustic environment, which has been shown to affect marine mammals and fishes. Invertebrates, such as bivalves, have so far received limited attention despite their important role in the marine ecosystem. Several studies have examined the impact of sound on anti-predator behavior using simulated predators, but studies using live predators are scarce. In the current study, we examined the separate and combined effects of boat sound playback and predator cues of shore crabs (*Carcinus maenas*) on the behavior of mussels (*Mytilus* spp.). We examined the behavior of the mussels using a valve gape monitor and scored the behavior from the crabs in one of two types of predator test conditions from video footage to control for effects from potential, sound-induced variation in crab behavior. We found that mussels closed their valve gape during boat noise and with a crab in their tank, but also that the stimulus combination did not add up to an even smaller valve gape. The sound treatment did not affect the stimulus crabs, but the behavior of the crabs did affect the valve gape of the mussels. Future research is needed to examine whether these results stand in situ and whether valve closure due to sound has fitness consequences for mussels. The effects on the well-being of individual mussels from anthropogenic noise may be relevant for population dynamics in the context of pressure from other stressors, their role as an ecosystem engineer, and in the context of aquaculture.

## Teaser

We exposed mussels to different types of predator cues from shore crabs and playback of boat noise or ambient control. Mussels reduced their valve opening in response to boat noise and a free-ranging predator. Valve opening is an indicator of food intake and comparative impacts of predators and noise yields insight into fitness consequences on mussels.

## INTRODUCTION

Increasing amounts of noisy human activities at sea have changed the underwater soundscapes of oceans and seas substantially over the past decades (Andrew et al. 2002; Hildebrand 2009; Sertlek et al. 2019). Many marine animals rely heavily on acoustics for information about their environment (Fay 2009; Slabbekoorn et al. 2010; Erbe et al. 2016). Anthropogenic noise, therefore, has the potential to affect the behavior and physiology of marine animals and affect their survival and reproductive success (Kunc et al. 2016; Slabbekoorn et al. 2018; Mills et al. 2020; Duarte et al. 2021). Invertebrates fulfill an important role in marine food webs and often provide critical ecosystem services (Morley et al. 2014; Solan et al. 2016). Nevertheless, sound impact studies are mostly focused on marine mammals and fish, whereas invertebrates have received far less attention (Williams et al. 2015; Di Franco et al. 2020; Duarte et al. 2021).

Several studies examined the effects of elevated noise levels on invertebrates. For crustaceans and bivalves, anthropogenic noise has been shown to change oxygen consumption (Regnault and Lagardère 1983; Wale et al. 2013a, 2019), increase biochemical stress parameters (Filiciotto et al. 2016; Vazzana et al. 2016), reduce growth and development (Lagardère 1982; de Soto et al. 2013; Nedelec et al. 2014), and even increase mortality (Nedelec et al. 2014; Day et al. 2017). Behaviorally, anthropogenic noise has been shown to change locomotor activity (Filiciotto et al. 2014; Filiciotto et al. 2016), disrupt feeding and change feeding rate (Wale et al. 2013b; Spiga et al. 2016; Hubert et al. 2018), and delay predator response (Chan et al. 2010; Wale et al. 2013b).

Sound impact is often studied in a single isolated species. However, *in situ*, other species may also be affected by the sound and thereby indirectly also affect the species of interest. For example, noisy gas extraction sites led to a decline in species richness in avian communities including the major nest predator, the western scrub-jay (*Aphelocoma californica*), which resulted in a higher breeding success for other species when compared with relatively quiet gas extraction sites (Francis et al. 2009; Slabbekoorn and Halfwerk 2009). In the lab, music from AC/DC reduced the foraging rate of lady beetle larvae (*Harmonia axyridis*) which led to higher numbers of soybean aphids (*Aphis glycines*), which in turn led to lower soybean plant (*Glycine max*) biomass. Without lady beetle larvae, the music did not affect aphid numbers and plant biomass (Barton et al. 2018). Another field study showed that fewer shore crabs (*Carcinus maenas*) came to a food item during experimental sound exposures, and this led to higher common shrimp (*Crangon crangon*) numbers due to competitive release (Hubert et al. 2018). This indicates that studying the effects of stressors on a single species may not be indicative of the effects *in situ*.

Several studies focused on sound-induced changes in anti-predator behavior, because such changes have the potential to impact survival directly. Elevated noise levels typically lead to a delayed or milder anti-predator response (Chan et al. 2010; Simpson et al. 2014; Morris-Drake et al. 2016; Simpson et al. 2016; McCormick et al. 2018; Kok et al. 2021), and occasionally animals may respond faster (Voellmy et al. 2014). Predators may not only directly affect survival, but the mere presence of predators may also yield non-consumptive effects (Lima and Dill 1989). Increased vigilance may make animals shift from spending time on feeding to scanning the environment, with potential fitness consequences (Kern and Radford 2016; Boudreau et al. 2018). A famous example of such non-consumptive effects is the change in foraging behavior and stress levels in elks (*Cervus elaphus*), after the reintroduction of wolves (*Canis lupus*) in Yellowstone National Park. The wolves not only trimmed the population directly by predation, but non-consumptive effects also lead to fewer calves (and cascading effects on the entire ecosystem: Creel et al., 2009; Ripple and Beschta, 2012). It may well be that such non-consumptive effects of predators are amplified by disturbance from anthropogenic noise.

The combined effect of multiple stressors does not necessarily equal the sum of the individual effects of the stressors, but can lead to “ecological surprises” (Hale et al. 2017). For example, heat stress and toxic metal (cadmium) combined lead to lower mitochondrial ATP synthesis and higher mortality in oysters (*Crassostrea virginica*) than the separate effects combined (Lannig et al. 2006). Conceptually, multiple stressors can be expected to result in (1) an additive interaction, the sum of individual effects, (2) antagonistic interaction, a weaker effect than the sum of the individual effects, or (3) a synergistic interaction, a larger effect than the sum of the individual effects (cf. Piggott et al., 2015 if individual effects are not in the same direction). A meta-analysis and later reanalysis, based on 170 studies on the effects of separate and combined stressors in marine ecosystems, showed that most studies reveal an antagonistic interaction, followed by synergistic, and lastly additive interactions (Crain et al. 2008; Piggott et al. 2015). Because ecosystems are complex environments full of stimuli, results of studies examining the effects of a single stressor are not necessarily a good predictor of the effects *in situ*. More studies with multiple stressors are needed to predict and understand the effects of disturbance in the field more accurately.

Mussels (*Mytilus* spp.) and shore crabs (*Carcinus maenas*) provide a nice model system to study the effects of combined predator presence and anthropogenic noise. Both species co-occur in the coastal waters of Europe and North America, and shore crabs and other mussels from the *Mytilus* genus can even be found around the globe (Hilbish et al. 2000; Carlton and Cohen 2003). The shore crab is an important predator of the mussel and predates it by crushing and breaking the mussels’ shell (Elner 1978; Capelle et al. 2016). Blue mussels have been shown to close their valves upon (simulated predatory) tactile cues (Clements et al. 2021), and develop thicker shells in response to chemical cues from shore crabs, leading to longer handling times for the crabs (Reimer and Harms-Ringdahl 2001; Freeman 2007; Freeman et al. 2009). Both mussels and crabs have been shown to respond to sound (Wale et al. 2013b; Hubert, Booms, et al. 2022) and are expected to be sensitive to the particle motion component of sound (Ellers 1995; Zhadan 2005; Edmonds et al. 2016). The hearing range of both species is not fully known yet, but blue mussels have been shown to respond to tones from 5 to 410 Hz (no tones outside this range used, Roberts et al., 2015). No such study exists for shore crabs yet, but mud crabs (*Panopeus* spp.), from the same infraorder as shore crabs, have been shown to detect sound from 75 Hz with decreasing sensitivity up to 1600 Hz (no tones outside this range tested: Hughes et al., 2014).

In the current study, we examined the effect of boat sound playbacks and the presence of shore crabs (predators) on the behavior of mussels (crab prey). In addition to a no-crab control, we exposed the mussels to crab presence (“stimulus crab”) in two conditions: exclusively chemical cues, or also potential visual and tactile cues from a free-ranging crab. We automatically logged the valve gape of the mussels with a valve gape monitor and manually scored the behavior and distance to the mussel of the stimulus crabs in the free-ranging predator conditions. We aimed to answer the following questions: (1) Does boat sound affect the valve gape behavior of mussels? (2) Does crab presence affect the valve gape behavior of mussels and are chemical stimuli sufficient for this effect? And (3), does boat sound affect the behavior of the stimulus crabs, and thereby a possible impact of the crabs on the mussels?

## MATERIALS AND METHODS

### Subjects

We used 149 mussels (*Mytilus* spp.) of 3.1–5.4 cm in length and 16 shore crabs (*Carcinus maenas*) with carapace widths of 1.5–4.4 cm in this study. The mussel study population probably consisted of pure *M. edulis*, hybrids with *M. galloprovincialis*, and occasional hybrids with *M. trossulus* (Coolen et al. 2020). The hybrids are not easily discriminated from pure *M. edulis* mussels, and molecular techniques are needed to do this. The animals were collected throughout April–June 2021 from the breakwaters of the fishing harbor in The Hague, The Netherlands. Both the mussels and crabs were kept at the Leiden University saltwater facility in separate stock tanks (185 × 40 × 50 cm; *L* × *W* × *H*), with continuously filtered water of 18–20°C at a 13 L: 11 D schedule. They were housed in stock for at least 24 h before being used in the experiment. The mussels in stock were fed with live phytoplankton (*Nannochloropsis* spp., Colombo), and the crabs were fed with dead mussels, both five times per week. The experiments were conducted in May and June 2021.

### Experimental setup and design

**Figure 1:**
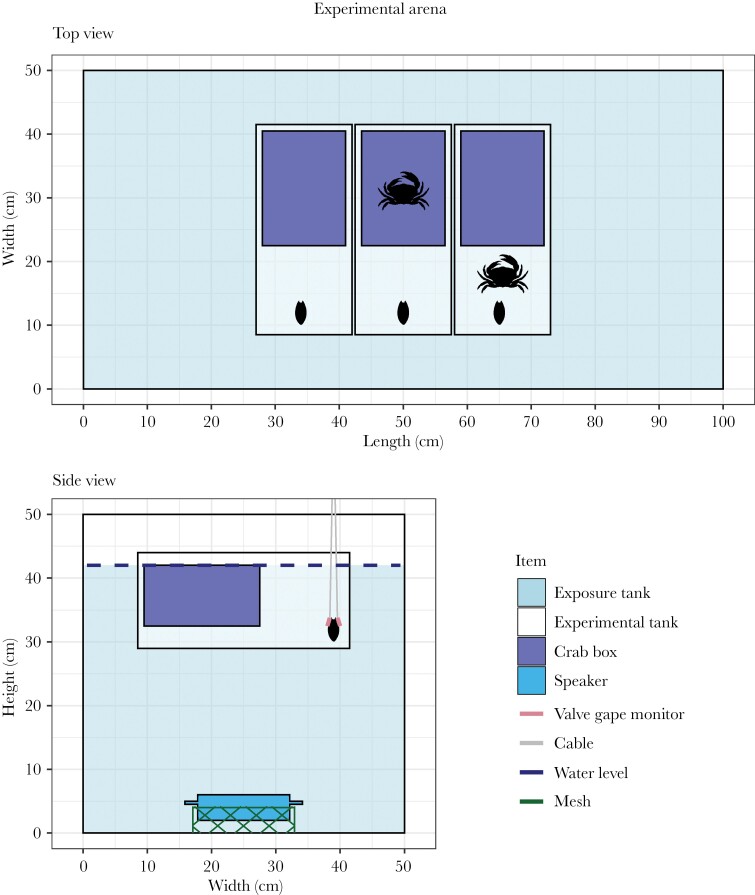
Schematic views of the experimental arena from above and aside. The mussels were hung in experimental tanks within a larger exposure tank, and exposed using a speaker at the bottom of the exposure tank. In each trial, we independently tested three mussels and we always tested three different crab conditions simultaneously: depicted from left to right (top figure) (1) no crab; (2) crab in box; (3) crab in tank. The silhouettes of the crab and mussel are drawn larger than to scale.

The trials were performed in a glass exposure tank (100 × 50 × 50 cm; *L* × *W* × *H*) with salt water and an underwater speaker on a mesh frame at the center of the tank floor. Above the speaker, and 13 cm below the surface, we hung three experimental tanks of polycarbonate (15 × 33 × 15 cm). Each experimental tank held a plastic box (13 × 18 × 10 cm) that could hold a live stimulus crab. The box had 24 small holes (4 mm ⌀) that allowed water flow and the exchange of chemical cues with the rest of the experimental tank (Figure 1). During each trial, we independently tested three mussels (one in each experimental tank), and exposed each individual to different crab (predator) exposure conditions: (1) “no crab,” with no predator cues; (2) “crab in box,” with chemical cues from a nearby live crab; and (3) “crab in tank,” with chemical and potentially visual, and tactile cues from a live crab in the experimental tank. We used black plastic between the experimental tanks to ensure independence of the trials. The valve gape behavior of the mussels was measured using a valve gape monitor and the crabs in the tanks were filmed (GoPro HERO 4) to be able to score their behavior.

**Figure 2:**
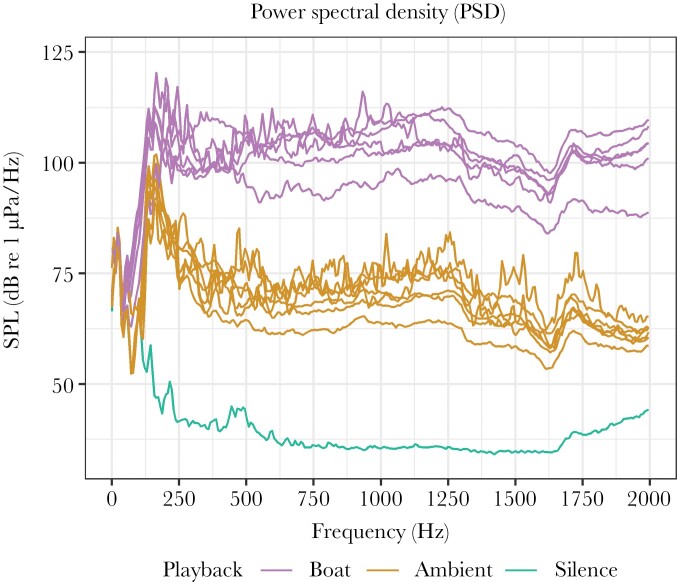
Power spectral density (window length: 6144, window type: Hann) of 30 s recordings of all boat and ambient stimuli, and the silence playback (tank ambient). The recordings were made in the center of the middle experimental tank at 10 cm below the water surface.

**Figure 3:**
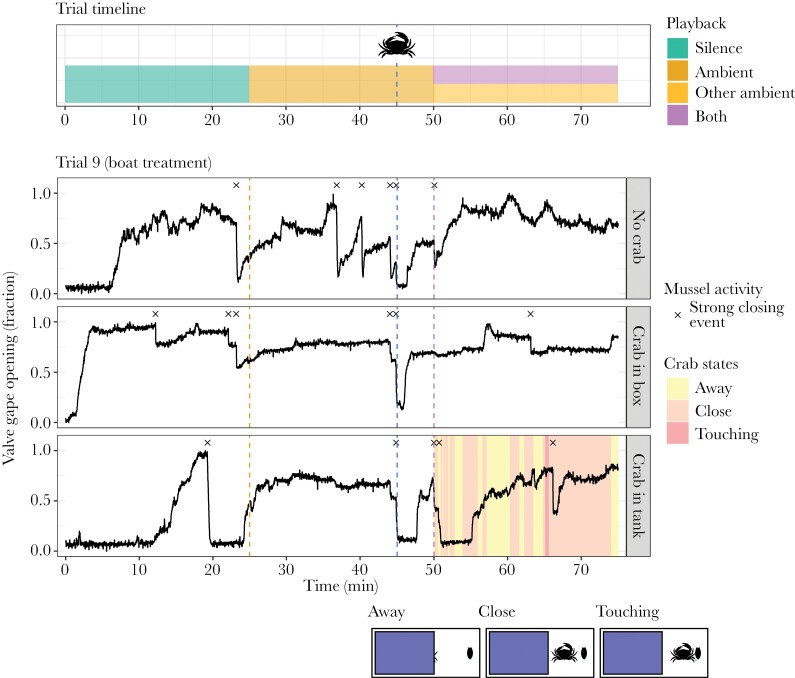
The timeline of a trial (top panel) and the data of an example trial (three bottom panels). Each trial started with 25 min of silence playback, followed by 25 min ambient playback, and consequently followed by 25 min playback of either boat or other ambient sound. The crabs were introduced (Crab in box & Crab in tank) or crab box was closed (No crab) 5 min before the boat or other ambient sound. The mussels typically opened up after the start of the experiment (and associated handling) and temporarily closed again (completely or partially) due to the introduction of the crabs and/or the closing of the crab box lids as control. The dashed lines indicate the onset of the ambient sound, introduction of the crabs, and the onset of the boat or other ambient sound (in this trial: ambient sound). The x’s indicate strong and fast valve gape closing events, and the colors in the “crab in tank” condition indicate the state of the stimulus crab (manually scored from video).

After starting the video recording and attaching the valve gape monitor on all three mussels, the individuals were hung in one of the experimental tanks at ~ 10 cm below the water surface and the trial was initiated. The trials lasted for 75 min and started with 25 min of silence, followed by 25 min of ambient sound, and followed by either another ambient sound or continuous boat sound (treatment) (Figures 2 and 3). We added the crabs 45 min after the start of the trials (5 min before the boat or second ambient sound) and closed the lids of the crab boxes in all experimental tanks to control for the effects of disturbance from the experimenter on the mussels. We decided for a relatively short period (5 min) between the crab introduction and sound onset to prevent habituation of the mussels to the crab stimulus. After each trial, the mussels were moved to a separate stock tank, the crabs were returned to their original stock tank, and the water in the experimental tank was refreshed. Assigning the crab conditions across experimental tanks was counterbalanced between trials, and each crab was used multiple times.

### Sound stimuli

The playback tracks were constructed using seven ambient recordings and six boat (3–~30 min long) recordings from our previous study (cf. Hubert et al., 2021). The original ambient and boat recordings were 35–50 s long and had a relatively constant amplitude and were first looped into 25 min tracks using 3 s linear crossfades. The 25 min ambient track was linearly crossfaded in 10 s to 25 min of either another ambient sound or continuous boat sound. We used 59 unique combinations of exposure stimuli for a total number of 59 trials. We band passed the tracks with 100–1600 Hz filters to anticipate on low-frequency speaker limitations and based on the expected high-frequency hearing limits of blue mussels and shore crabs (Hughes et al. 2014; Roberts et al. 2015). The sound treatments were created using a combination of R (R Core Team 2016) and Audacity (version 2.4.2) and played back with a UW30, Lubell labs underwater speaker from a DR-05, TASCAM recorder, through an M033N, Kemo amplifier. Trials with ambient and boat treatments were alternated, nine of the trial days started with an ambient treatment and eight with a boat treatment, the specific tracks were randomly selected.

Between the trials, we recorded 30 s pieces of all sound treatments with a calibrated hydrophone (96-min, HTI) and digital recorder (DR-100MKII, TASCAM) in the center of the central experimental tank, at 10 cm below the water surface. We generated power spectral density plots using a custom-made R-package (Figure 2). The rms SPL (sound pressure level, geometric mean of all locations in the 100-600 Hz bandwidth) was 145.4 dB re 1 μPa for the boat stimuli, 125.4 dB re 1 μPa for the ambient stimuli, and 92.4 dB re 1 μPa for the silence playback. Mussels and crabs are probably solely sensitive to particle motion rather than sound pressure, so ideally, we would have measured this instead. However, we did not have a small enough sensor that would have been able to measure particle motion at such a small spatial scale. We used the sound pressure measurements to show that sound of the given spectrum was present and to make the exposure reproducible.

The sound propagation in the experimental tanks can be expected to differ substantially from the propagation of actual anthropogenic sound at sea. The proximity of the tank walls and water surface affects the ratio between sound pressure and particle motion, and the directionality patterns of particle motion (Rogers et al. 2016; Campbell et al. 2019). For this reason, we used experimental tanks within a larger exposure tank, but this will only limit the mentioned effects at the position of the study subject to some extent. Additionally, a speaker – which we used for the playbacks – is a point source whereas a boat is not and the same applies to many ambient sound sources (Wales and Heitmeyer 2002). However, the artificial acoustic field of our test conditions does not pose a problem for the current proof-of-concept study as we aimed to gain insight into the behavioral interaction between crabs and mussels and its potential impact on the type and magnitude of the response to a particular acoustic stimulus, rather than determining absolute response levels to boats that are representative for crabs and mussels *in situ*.

### Valve gape monitor

We used a valve gape monitor to log the valve gape behavior of the mussels, the same monitor as we used in our previous study on mussels (Hubert, Booms, et al. 2022). The valve gape monitor consisted of multiple pairs of electromagnetic coils. The active coil of each pair generated an electromagnetic field, which resulted in a current in the responsive coil. We attached the coils of one pair on opposite valves of an individual mussel using hot glue in combination with cyanoacrylate glue. The strength of the measured electromagnetic field was determined by the distance between the coils, and thus reflected valve gape. The valve gape monitor yielded on average 47.3 (range: 47.1–47.6) datapoints per minute for each individual. The measured electromagnetic field strength was converted to absolute distances between the sensors using the calibration of the monitor. By subtracting the minimum distance of each mussel from all measured distances of the same mussel, we determined the valve gape (at the location of the sensors). To account for inter-individual variation in size and the location of the sensors, we subsequently normalized the data by dividing the valve gape by the maximum valve gape of this individual to obtain the fraction open.

We determined the short-term response of the mussels to the change in playback (at 50 min) by calculating the difference in mean valve gape between 30 s before and 30 s after the sound onset (in accordance with Hubert, Booms, et al. 2022). We determined the long-term response by calculating the difference in mean valve gape before the crab introduction (min 40 to min 44) with the mean valve gape after the onset of the sound (min 50 to min 75). By taking into account such baseline data, we accounted for inter-individual variation in valve gape levels. Additionally, we automatically detected sudden and strong closing events. For this, we used a 15-datapoint moving average of the valve gape data and calculated the difference of sequential moving average datapoints (ΔMA). As a threshold for strong closing events, we used −15 times the standard deviation of the 50% of the ΔMA-data around its median. We determined the difference in rate (strong closing events per 10 min) before the crab introduction (20.5–44 min) with the rate during the experimental sound (50.5–75 min), we kept a 30 s margin after the sound onset to exclude inclusion of closing events due to the sound onset. We performed 59 trials with 177 mussels, 28 individuals were excluded because of sensor failure or crab escapees.

### Video recordings

The experimental tanks were filmed from above to be able to score the behavior of the stimulus crab in the experimental tank. The second author scored the videos of the last 25 min of each trial while being blind to the sound treatment (either ambient or boat sound). All timestamps of switches between the following states of the crab were scored: (1) Staying under the crab box, so being relatively far “away” from the mussel; (2) Not staying under the crab box, so being relatively “close” to the mussel; (3) “Touching” the mussel (Figure 3). We used the timestamps to determine the fraction of time spent in each of the three states. We managed to obtain and score videos for 49 out of the 54 trials with mussel data from the crab in tank condition, the camera stopped working during the other 5 trials. The current experiment was not designed to study the effect of boat sound on crabs, as we used the stimulus crabs repeatedly. However, we quantified the behavior of the crabs to determine whether potential differences in mussel behavior during sound treatments, could be explained by sound-induced differences in the behavior of the stimulus crabs.

### Data processing and statistics

We analyzed the effect of the sound treatments and crab stimulus conditions on the change in valve gape (Δ fraction open), strong closing events, and the separate behavioral states of the stimulus crabs. We used generalized linear models (GLMs) with a Gaussian error distribution and identity link-function, except for the time spent touching the mussels by the stimulus crabs, for which we used a Poisson error distribution with log link-function. When applicable, we used the sound treatment, crab stimulus condition, the interaction between sound and crab condition, the size of the mussel, and the size of the crab into the full model. For each full model, we determined the AICc score of all possible explanatory variable combinations and selected the model with the lowest AICc as the best model. To determine the effect and significance of the covariates, we ran the best models. To make pairwise comparisons between the crab conditions, we used a Tukey’s posthoc test. The interaction between sound treatment and crab stimulus condition was never part of the best model, potentially the sample size did not allow for an integrated analysis. To gain insight into potentially changing effects of sound due to the crab stimulus anyway, we made separate models to evaluate the effect of sound per crab condition when examining the long-term effects of sound and crab. All data processing, plotting, and analyses were done using R (R Core Team 2016) and the packages ggplot2 (Wickham 2016), MuMIn (Barton 2016), and multcomp (Hothorn et al. 2008).

## RESULTS

**Figure 4:**
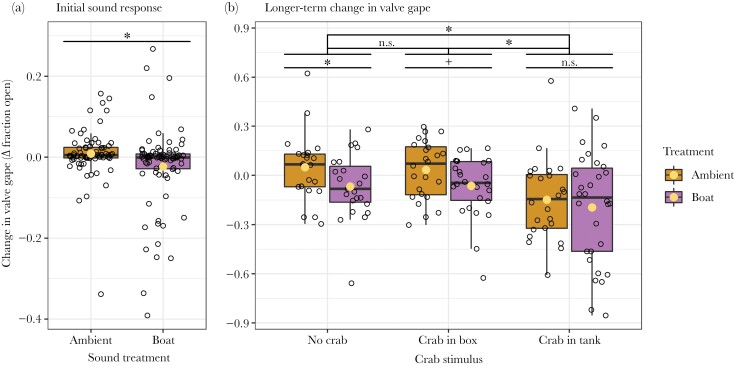
Short-term and long-term effects of sound treatment and crab stimulus condition on mussels’ valve gape. (a) The initial response is determined by the difference in valve gape between 30 s before and after the change in sound stimulus (at 50 min). Mussels in the boat condition had a significantly lower Δ valve gape (a decrease) when compared with the mussels in ambient conditions (b) The long-term change in valve gape is reflected by the difference in valve gape between a 4 min period before the introduction of the crab and the 25 min of sound treatment. Irrespective of sound treatment, the mussels in the crab in tank condition had a lower Δ valve gape than the other two crab conditions. Overall, mussels that were exposed to boat sound were more closed. When examining the crab conditions separately, we found that mussels in the no crab condition that were exposed to boat playback had a significantly lower Δ fraction open. For the mussels in the crab in box condition, this was only a non-significant trend. There was no difference in the change of valve gape between sound treatments for the mussels in the crab in tank condition. The box-and-whisker plots indicate the median, first, and third quartile and, minimum and maximum, excluding outliers of all individuals per exposure. The open circles indicate the individual mussels and the light-yellow circles indicate the means. The asterisks (*) indicate significant differences (*P* < 0.05), the plus (+) indicates a non-significant trend (*P* ≥ 0.05 < 0.10), and “n.s.” indicates a non-significant difference (*P* ≥ 0.10). Note that the scale of the y-axes differs between the plots.

Mussels that were exposed to the boat sound treatment (from 50 min onwards) initially responded to the boat sound by partially closing their valves relative to the mussels in the ambient condition (intercept: 0.058, Boat: −0.031, *P* = 0.03; Figure 4a). The crab stimulus condition or its interaction with sound treatment was not part of the best model on the initial response to the sound treatment. Over the entire sound treatment period and when comparing different crab conditions, there was no difference in the mussels’ valve gape in the no crab and crab in box condition (no crab – crab in box: 0.006, *P* = 0.99; Figure 4b), but the mussels’ valve gape was smaller in the crab in tank condition than in the no crab and crab in box condition (no crab – crab in tank: 0.160, *P* < 0.01; crab in box – crab in tank: 0.154, *P* < 0.01; Figure 4b). Across crab conditions, mussels were more closed during boat sound treatment (intercept: 0.033, boat: −0.085, *P* = 0.03; Figure 4b). The interaction between sound and crab condition was not part of the best model, even though examination of the visual representation of the data suggests that boat sound does not have the same effect across crab conditions (Figure 4b). For this reason, we also examined the effect of sound per crab condition separately. Mussels in the no crab condition that were exposed to boat playback had a smaller valve gape than mussels that were exposed to ambient playback (Intercept: 0.697, Boat: −0.128, *P*-value: 0.04; Figure 4b). In the crab in box condition, there was a non-significant trend that mussels exposed to boats were also less open (Intercept: 0.034, Boat: −0.097, *P*-value: 0.07; Figure 4b). In the crab in tank condition, there was no difference in valve gape between playbacks (Intercept: −0.148, Boat: −0.047, *P*-value: 0.57; Figure 4b).

**Figure 5:**
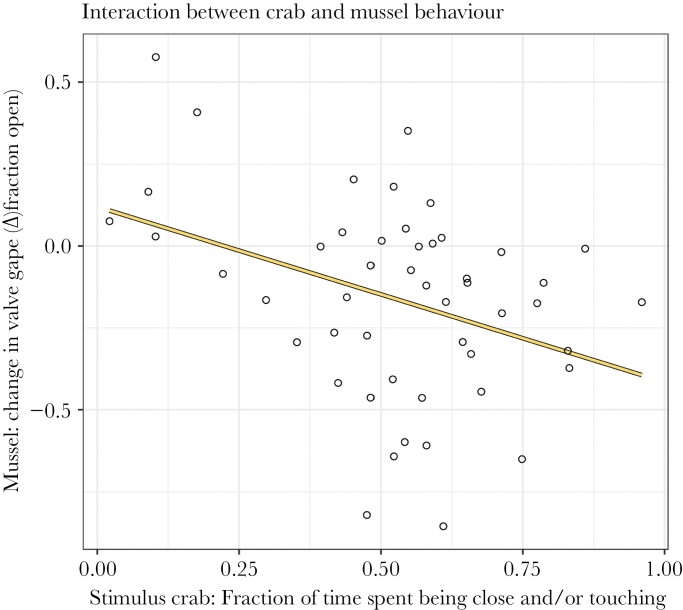
A significantly negative correlation between the valve gape of mussels and the time spent by the crab being close to the mussel and/or touching it. The sound treatment covariate was not part of the best model and did not affect this correlation. Just like Fig. 4b, the mussels’ valve gape is reflected by the difference in valve gape before the introduction of the crab and during the sound treatment. Data is from mussels and crabs in the crab in tank condition only. Each open circle indicates a trial and the yellow solid line indicates the significant trend.

**Figure 6:**
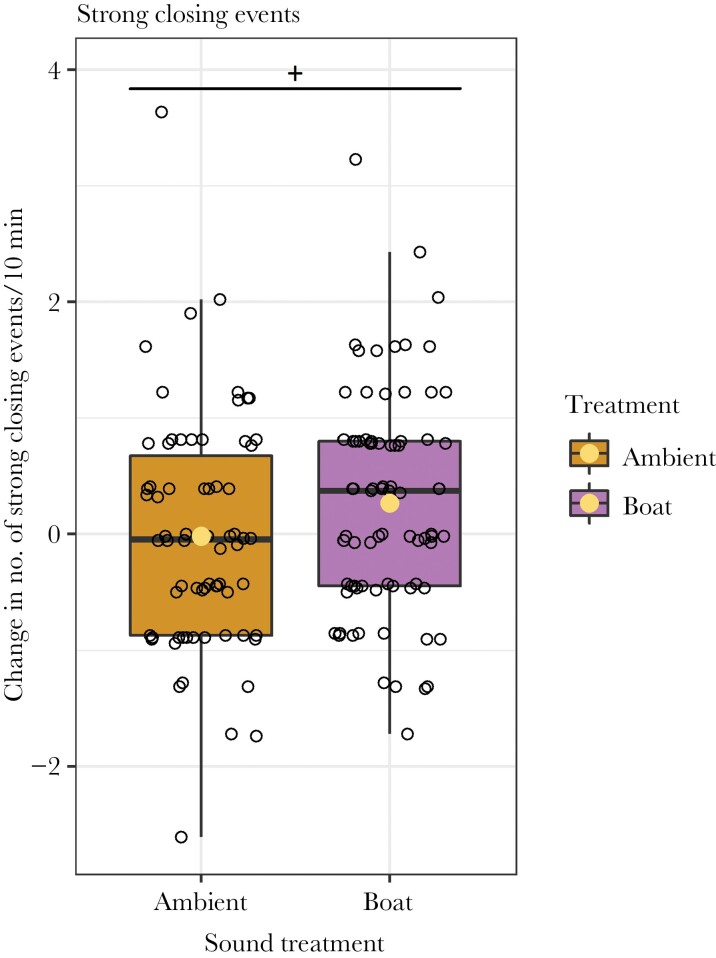
Change in the amount of sudden and strong closing events in the mussels’ valve gape. The change in closing events is a result from comparing the 23.5 min before the crab introduction (1 min margin before crab introduction), and the 24.5 min after the onset of the sound (0.5 min margin after sound switch). A non-significant trend (+) indicated that mussels that were exposed to boat playback increased the number of strong closing events more than the mussels that were exposed to ambient sound.

We found that valve gape was negatively correlated with the time crabs were close to the mussel and/or touching the mussel in the crab in tank condition (Intercept: 0.120, slope: −0.535, *P*-value < 0.01; Figure 5). A non-significant trend indicated that mussels across crab conditions showed slightly more immediate closing events during boat playbacks (Intercept: −0.017, Boat: 0.284, *P*-value: 0.07; Figure 6).

**Figure 7:**
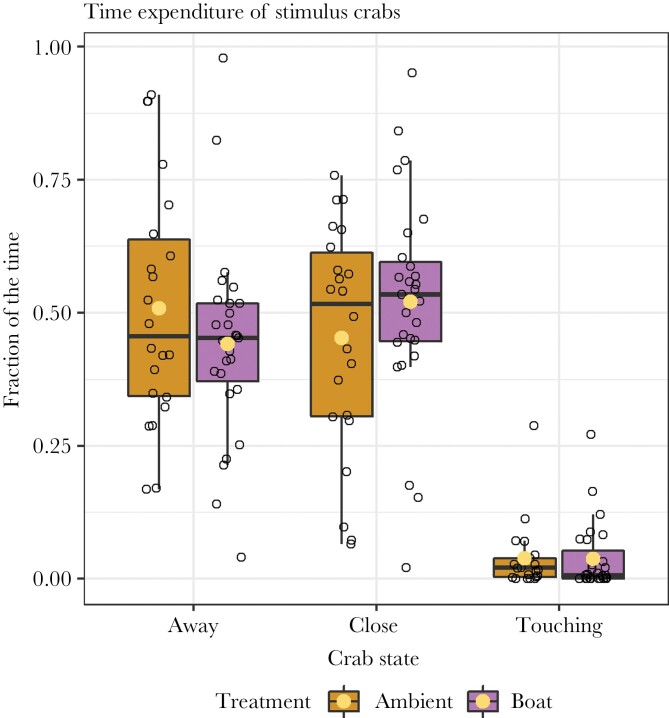
Time expenditure of the stimulus crabs being away, close, or touching the mussel, only for the crabs in the crab in tank condition. The crab state was scored in the last 25 min of the trials (5 min after crab introduction). We found no differences in time expenditure between crabs in the ambient and boat conditions. Crabs were used repeatedly.

We found no significant differences in the time expenditure of stimulus crabs in the crab in tank condition due to the sound treatment. The crabs were similar times away from the mussel (Intercept: 0.509, Boat: −0.067, *P*-value: 0.26; Figure 7), close to the mussel (Intercept: 0.453, Boat: 0.068, *P*-value: 0.26; Figure 7), and touching the mussel (Intercept: −3.267, Boat: −0.013, *P*-value: 0.99; Figure 7). This indicates that differences in mussel behavior between sound treatment can likely be explained as direct effects of the sound on the mussel, rather than an indirect effect through sound treatment induced differences in crab behavior.

## DISCUSSION

In the current study, we exposed mussels to the playback of boat sound or an ambient control shortly after the start of partial or full exposure to predator cues from a nearby shore crab. Our results demonstrate that mussels closed their valves upon the onset of the boat sound. The mussels remained more closed during the boat sound exposure than during ambient noise, which was significant without crab stimulus, a non-significant trend with the chemical presence of a crab in a box nearby, but absent with a crab in the same tank as the mussel. This was because regardless of the sound treatment, the mussels were less open with a crab in the same tank than in the other conditions. The time that the crabs in the tank spent close to the mussel or touching it, was negatively correlated with the relative valve gape of the mussels. We found no evidence for an effect of the used sounds on the repeatedly used stimulus crabs, indicating that the crab stimulus was similar across sound treatments.

### Sound effects on mussels

The mussels responded to the onset of the boat playback by closing their valves and – without stimulus crab – remained more closed during the entire 25 min exposure. Previous studies have already shown a short-term valve closure response to pure tones (with harmonics) (Roberts et al. 2015; Hubert, Booms, et al. 2022; Hubert, Moens, et al. 2022). In the current study, we used broadband boat noise playback which started with a 10 s linear cross-fade from ambient sound; essentially a 10 s fade in, but the mussels still responded. An earlier study on fish also showed that a soft start not necessarily diminishes the response to sound (Neo et al. 2016). The relatively long-term, more-closed valves of the mussels in the current study are in contrast with another study that found a larger valve gape during 1 h exposure to ship noise (Wale et al. 2019). The latter study only tested a limited number of individuals, but part of the discrepancy may also be explained by the longer duration of that experiment, as the response of mussels to sound has been shown to diminish over repeated sound exposures (Hubert, Booms, et al. 2022) and to return or exceed baseline levels during extended series of impulsive sound exposures (Hubert, Moens, et al. 2022).

Valve closure in mussels is also reported in response to fluctuations in temperature and salinity, air exposure (Bayne et al. 1976), potentially poisonous chemicals (Curtis et al. 2000), conspecific homogenate (Robson et al. 2010), tactile cues (Clements et al. 2021), and reduced food availability (Mills and Reynolds 2004). In the field, Eastern oysters (*Crassostrea virginica*) have also been described to close their valves due to predator (blue crabs, *Callinectes sapidus*) presence (Carroll and Clements 2019). (Partial) valve closure will therefore likely serve in the protection of the mussels’ tissue from adverse environmental conditions and predation (Robson et al. 2010), but at the same time may also reduce the feeding rate of mussels. Although dissociation also occurs (Maire et al. 2007), previous experiments demonstrated that valve gape is linked to feeding rate (Jørgensen et al. 1988; Hubert, Moens, et al. 2022).

A non-significant trend also indicated that mussels exhibited more sudden and strong closing events during the boat playback. Previous studies have shown that mussels regularly close rapidly, although they typically open gradually (Wilson et al. 2005; Robson et al. 2007). Rapid closure has been linked to both the elimination of faeces and pseudo-feces as to potential danger (Gosling 2003; Robson et al. 2007). It may be an indication of increased vigilance, as noise-induced increased vigilance has been found in fishes (Voellmy et al. 2014), birds (Quinn et al. 2006), and mammals (Kern and Radford 2016). The energy consumption for a mussel to open or remain closed is expected to be limited, but rapidly closing is not (Galler et al. 2010; Robson et al. 2010). This indicates that increased vigilance resulting in more closing events could not only reduce food intake, but also be energetically costly.

### Crab stimulus effects on mussels

Mussels with a stimulus crab in the same tank had the smallest mean relative valve gape when compared with mussels without crab or with a crab in a box. There were no differences between the latter two conditions. In the condition with the stimulus crab in a box, we expected the crab to excrete chemical cues and – depending on their activity – acoustic and vibration cues. The latter two cues may have been relatively unnatural because the crab was in a mid-water plastic container rather than on a sandy, hard-substrate, or reef seafloor. Either way, the chemical (and acoustic/vibration) cues were not sufficient to elicit a significant change in valve gape behavior of the mussels. This is in contrast to our expectations because mussels have been shown to close their valves due to conspecific homogenate, another chemical cue indicating predation (Robson et al. 2010; Clements et al. 2020), and oysters have also been observed to close their valves in the chemical presence of crab predators. It may be that the number of holes in the plastic box and their size was too small, potentially in combination with the limited movement of the crab, to generate enough flow of chemical cues. It may also be that mussels are not very sensitive to such chemical shore crab cues. Conspecific homogenate to elicit an anti-predator response may be easier to control in terms of concentration and release location. In the condition with the stimulus crab in the same tank as the mussel, the crab was expected to provide chemical and potentially tactile, visual, and acoustic/vibration cues. The chemical and acoustic/vibration cues were likely stronger than in the condition with the crab in the box. Here, the combination and/or level of cues was sufficient to elicit a strong anti-predator response. This is in line with the field observation that oysters closed their valves due to the presence of predatory crabs (Carroll and Clements 2019).

When the crab was in the same tank as the mussel, there was a negative correlation between the valve gape of the mussel and the time the crab was close to the mussel and/or touching it, irrespective of the sound treatment. It is not possible to definitively conclude whether crabs were more likely to approach the mussel because the mussel had a smaller valve gape, or whether the mussel had a smaller valve gape because the crab was nearby. However, mussels have been shown to close in response to a chemical indication of predation (conspecific homogenate), and closed valves make it harder for crabs to eat or detect the prey, however, experimental evidence is scarce (Norton-Griffiths 1966; Robson et al. 2010; Wilson et al. 2012). Based on this, we assume the mussels responded to the crabs and not vice versa.

### Noise and crab stimulus combined

Without stimulus crab, the mussels closed their valves more during boat sound when compared with ambient sound. For the mussels with a crab in a box, this was a non-significant trend, but with a crab in the same tank, this effect was completely absent. This is an example of an antagonistic interaction, where two stressors have a lower impact than the sum of the two separate effects (Hale et al. 2017). There are several potential explanations for why we did not find an additive or synergistic effect of the sound: (1) the crab behaved differently because of the sound; (2) the mussel already exhibited a maximum anti-predator response because of the crab stimulus and could not close further; (3) there was no need for the mussel to respond to the sound anymore because it was already partly closed because of the crab stimulus; (4) the predator or noise stimulus (partly) masked the other stimulus; (5) the combination of predatory cues and noise hindered the processing or interpretation of both stimuli (Halfwerk and Slabbekoorn 2015; Hubert et al. 2021). The first explanation is not very likely because we found no difference in crab behavior due to the sound. The second explanation is also not very likely because the mussels were not closed completely whereas they can remain fully closed for hours, e.g., because of the tide (Andrade et al. 2018). Testing hypothesis 3 against 4 and 5 are needed to determine whether the lack of response to the sound is maladaptive or not.

Our results show anyway that the anti-predator behavior of mussels to shore crabs was still present during noisy conditions, and that the combination of noise and a predator—that both elicit valve closure—had no additive effect and did not elicit an extra strong response. Previous studies have shown that sound can change the behavior of one species, and thereby indirectly also affect other species (Francis et al. 2009; Barton et al. 2018; Hubert et al. 2018). Such effects have not been found here, maybe because the predator was not affected by the sound, or because the behavioral response of the mussel to sound was of similar nature as their anti-predator response.

## CONCLUSIONS

The current study examined the separate and combined effects of boat sound playbacks and predator cues of a shore crab on the valve gape behavior of mussels. Both, boat sound and crab presence in the same tank as the mussel, resulted in a reduced valve gape. The stimuli combined did not lead to a larger effect than the predator alone, and predator alone led to a stronger response than sound alone. We found no evidence that the behavior of stimulus shore crabs was affected by the sound, but the mussels were—irrespective of the sound treatment—affected by the behavior of the crab. Our findings suggest that potential masking of predatory cues by boat sound is not raising predation risk for mussels in the current captive test conditions, but that unnecessary closing by erroneous perception of predator presence or increased vigilance in general, may have detrimental consequences for mussels in terms of growth, maturation, and survival through reduced food intake and increased energy expenditure. Further research is needed to determine the mechanisms underlying the current results, and should examine whether these results may be relevant for population developments in the field in the context of pressure from other stressors, their role as reef builder and habitat formation, and in aquacultural context.

## Notes

We thank Peter C. Snelderwaard for technical support and help with the animal care. JH was funded by an NWA grant (NWA.1236.18.004) from the Netherlands Organization of Scientific Research (NWO).

## DATA ACCESSIBILITY

Analyses reported in this article can be reproduced using the data and scripts provided by Hubert et al. (2023).
